# ‘Tsatsu tsɛ̃ɛ̃ bo…’: Societal reactions to male infertility among the Ga’s in Ghana

**DOI:** 10.1371/journal.pone.0330529

**Published:** 2025-08-29

**Authors:** Rosemond Akpene Hiadzi, Godwin Banafo Akrong

**Affiliations:** 1 Sociology Department, University of Ghana, Accra, Ghana; 2 School of Information Studies, McGill University, Montreal, Canada; Faculty of Medicine Universitas Indonesia, INDONESIA

## Abstract

Patrilineality plays a significant role in determining and confirming an individual’s family membership based on their father’s lineage. However, despite the importance of patrilineality in some societies, male infertility—which contributes significantly to infertility and accounts for half of all cases—has emerged as a significant concern. Thus, the study explores societal reactions to male infertility in Krokobite, a suburb of Accra, which is a predominantly Ga-speaking and patrilineal society. The study adopted a qualitative approach, utilizing in-depth interviews (24), focus group discussions (9), and key informant interviews (3). The study’s findings primarily attribute infertility to women, identifying blood incompatibility between couples as a contributing factor in some situations. The findings additionally show that men who are unable to have children or who delay having children face persistent teasing from their peers, who seek to encourage them to join the group of “responsible” men in society by having a child. The study further concludes that the concept of reproductive masculinity is evident in how participants regard males as fertile and less prone to reproductive harm. However, it does not show a significant relationship between reproductive masculinity and the role of fathers in identifying the cause of infertility in a relationship.

## 1. Introduction

In the majority of clans in Ghana, patrilineality plays a significant role, in determining and confirming an individual’s family membership based on their father’s lineage. When this occurs, it also links the inheritance of property, privileges, surnames, or titles to those related through male kin [1]. Nevertheless, amidst the numerous advantages of patrilineal ethnic groups, certain disadvantages exist, such as the mounting pressure on men by the family. This is because, in patrilineal societies, men are often responsible for maintaining the family lineage and supporting their extended family members. Men may experience considerable societal pressure to meet certain standards, which can have a detrimental effect on their mental health and overall well-being [2]. Certain individuals also often go unnoticed in patrilineal societies that prioritize biological ties over non-biological ones. This includes adopted children and those with non-biological relationships with the family, as emphasized by Ampofo et al. [3] and Layefa et al. [4]. However, it is critical to acknowledge that elements such as cultural norms and economic realities frequently shape these issues regarding patrilineal society [5].

It is therefore vital to explore how patrilineal societies respond to infertile males at a time when male infertility has become a major worry for society and families facing such difficulties. Given that these societies value biological relationships more than non-biological ones, gaining insight into the societal reactions to male infertility within patrilineal societies would be of immense significance. In many patrilineal societies, men often face significant societal pressure to uphold certain standards that involve taking responsibility for preserving the family lineage. Nevertheless, few studies have explored the reactions of families who employ these men to ensure the continuation of their family lineage when these men are unable to have children [6,7]. Thus, it would be intriguing to explore how family members and siblings react to a male sibling who is unable to have children and how this affects marital relationships. Additionally, it is important to explore the dynamics of such relationships when a woman has a child outside of wedlock.

There is also a great level of concern about what happens to marital relationships that are unable to produce children in patrilinear society, as Mumtaz et al. [8] emphasized that gender ideology, beliefs, and expectations influence infertility experiences, potentially undermining marital relationships and carrying gendered implications. However, their study further argued that men’s social identity, security, and authority were unaffected by poor marital bonds, despite only modest teasing from friends. Luk and Loke [9] also acknowledged that infertility has an impact on couples in four specific areas of their lives: psychological well-being, marital relationships, sexual relationships, and overall quality of life. Their findings also indicate that there is evidence linking infertility to poor psychological well-being and unhealthy sexual relationships, although there are conflicting data regarding its effects on quality of life and marital relationships. Therefore, additional research is required to address this research gap.

To address this research gap, it is essential to explore the actions of married infertile men when faced with the inability to have children. Given that Sedziafa et al. [10] and Peters [11] show that males in patrilineal communities face significant pressure to continue their father’s lineage, this gap will help provide valuable insights into the consequences of infertility for marital relationships. Iwelumor et al. [12] explained that societal norms ingrain the importance of children and closely associate it with religion, patriarchy, and the necessity of a stable family and marriage. People considered children to be divine, viewing childbearing as the fulfillment of a divine command. Most males saw children as life itself, whereas women regarded them as sources of fulfillment and a balanced life. People often believe that boys safeguard the integrity of the family tree and confer dignity and status upon men. Dolan et al. [13] assert that societal norms that discourage weakness and vulnerability, along with men’s limited understanding of their own bodies, can hinder men’s desire to have children. These factors contribute to men’s reluctance to seek help, as they aim to avoid appearing weak and vulnerable. Dolan et al. also observe that infertile men’s bodies were both a failing entity (unable to father a child) and a subordinated social entity (unable to meet hegemonic norms), which shaped how men negotiated and enacted their identities as men. Thus, in taking responsibility for their infertility and actively supporting each other, men appeared eager to embrace subordinate male practices, but this did not completely unravel well-accepted masculinity. Ultimately, the illustration of men coping with the unpredictability of infertility further contended that they were unable to alleviate their own anxieties about the future and the possibility of their partners choosing to have a child with someone else.

Typically, couples make significant efforts to preserve their marriages because it ultimately impacts their capacity to assume leadership positions within their families. According to a study by Tabong and Adongo [14], infertile couples face social stigma and are excluded from holding leadership roles in their communities. Additionally, couples without children are denied access to the ancestral world, resulting in the loss of the opportunity to be reborn. During this occurrence, both males and females participate in sexual activities with several partners as a means to demonstrate their reproductive capability. The existing literature indicates that there have been numerous studies conducted on the involvement of infertile women in assuming leadership roles within families, as well as their participation in various rites of passage [14,15]. However, there are only a few studies examining this from the perspective of infertile men. Therefore, it is necessary to fill this gap by conducting additional studies from the perspective of patrilineal societies. Culley et al. [16] emphasized the necessity for additional research involving men in various domains, such as the understanding of fertility and infertility, seeking treatment, undergoing treatment, information and support requirements, decisions to terminate treatment, post-assisted conception fatherhood, and the motivations and experiences of sperm donors and men pursuing fatherhood through surrogacy or co-parenting.

The current study argues that further research is necessary to thoroughly examine how society reacts to male infertility. Thus, the objective of the current study is to understand how patrilineal societies react to male infertility. To accomplish this, the current study relied on the Ghanaian Ga community, specifically Kokrobite. To achieve this objective, the researchers also looked at how siblings or family members respond to a sibling who is unable to have a child, what married men do when children are not forthcoming in their marriage, whether or not males can assume leadership roles in patrilineal families or communities if they do not have kids, and what happens to heads of families or lineages who are childless when it comes to carrying out various rites of passage.

## 2. Conceptual framework

To achieve the stated objective, the current study’s conceptual framework was based on reproductive masculinity. According to Daniels [17], the concept of reproductive masculinity is a set of beliefs and assumptions about men’s relationship to human reproduction. Four arguments in reproductive masculinity assert that while both men and women contribute essential genetic material to conception, women’s role in gestation, birth, and lactation presumably places males secondary in human reproduction. The second assumption is that men are less susceptible to reproductive harm than women. Third, men are virile, ideally capable of fathering their own biological children. However, although reproductive technologies and medical interventions make it possible for infertile men to become fathers, men’s infertility is still understudied, a source of personal shame, and shrouded in comparative secrecy. Fourth, men are relatively distant from the health problems of the children they father.

Therefore, the current study embraces this notion, identifying that most patrilineal societies hold the belief that men are less vulnerable to reproductive harm than women. Additionally, they presume that men possess virility, preferably having the ability to procreate with their own biological offspring. Although advancements in reproductive technology and medical procedures have enabled men who are unable to conceive naturally to achieve fatherhood, the present study utilizes this concept to thoroughly assess the dynamics between patrilineal community members and a man who is incapable of having children, as well as the reactions of family members and/or siblings towards a sibling who is unable to have children.

Furthermore, while men may benefit from the economic and social privileges associated with assumptions about reproductive differences, they also face significant consequences that may result in their losing their role as fathers or prominent players in their community [17]. This underscores the need to investigate whether men without children can assume leadership roles within their families or communities, as well as the circumstances surrounding the execution of various rites of passage by lineage or family heads who are childless in patrilineal societies.

It is significant to explore further the actions taken by men in patrilineal communities when they are unable to have children. The concept of reproductive masculinity is also based on the belief that being able to father biological children is crucial for a man’s social status [18]. Throughout history, this notion has resulted in a lack of attention towards male infertility and the belittlement of men who do not meet its expectations. Since fathering biological children is essential to one’s status as a man in a patrilineal society, the current study also examines what happens to relationships in patrilineal societies that cannot have children and what solutions are available to couples who cannot have children. Furthermore, the shame associated with male infertility has led to a tendency to hide this condition. Therefore, in the scenario where a woman has a child out of wedlock while hiding the infertility of her partner, what are the consequences for the marital relationship within a patrilineal society?

## 3. Methods

### 3.1. Study design

This study utilised a qualitative methodology approach consisting of individual in-depth interviews, Key informant interview, and focused group discussions as it allowed the study to understand how patrilineal cultures react to male infertility.

### 3.2. Study area

The study focuses on Kokrobite, a small town located along the Atlantic Coast to the west of Accra, the capital city of Ghana. The area lies within the Ga South municipality. According to the 2021 population and housing census, the total population in this area is 350,121, with 266,721 (76.2%) residing in urban areas and 83,400 (23.8%) in rural areas [19]. The 2021 population and housing census reported the urban population by counting the number of people in areas with a population of 5,000 or more. For the rural population, they counted the number of people in areas with less than 5,000. Kokrobite is renowned for traditional sea fishing, pristine sandy beaches, and vibrant nocturnal entertainment. The inhabitants of the area are also involved in small-scale farming for survival. Furthermore, the recent surge in coastal tourism in Kokrobite has led to an increase in the number of residents and migrants seeking employment in various businesses like bars, restaurants, hotels, and resorts. These individuals primarily work in roles such as cooks, caterers, waitresses, tour guides, handicraft manufacturers, laborers, and other types of unskilled positions [20]. Ga is the predominant local dialect; however, Twi and English are also often used [21]. The main reason Kokrobite was selected as the study region was because of its peri-urban location. In particular, it has characteristics that are evocative of rural as well as urban settings. Additionally, it was ideal since it exemplifies an indigenous community in the Greater Accra Region. Furthermore, the majority of the people who live in that area belong to the Ga ethnic group, who follow a patrilineal system of inheritance, whereby they inherit through their fathers. By choosing this specific area, the researchers were able to more effectively accomplish their objective, which is to investigate the perspectives of men residing in a patrilineal society in Ghana regarding male factor infertility.

### 3.3. Participants

The study participants included adult males residing in Kokrobite, Ghana, who were 18 years of age or older and were single, married, separated, or cohabiting. The study included participants who were both native speakers of the Ga language and those who identified as Ga. The snowball sampling approach was used to recruit these male participants for this study. This is because the snowball approach assists researchers in identifying new possible participants [22]. Nevertheless, the researchers meticulously executed the use of snowballing, taking into account the potential concern of sample bias. This is because, according to Sefcik et al. [23], participants can be more inclined to recommend those who have similar traits or experiences, which could distort the sample. Therefore, the present study employed many data collection strategies to verify the data gathered and enhance the trustworthiness of the findings.

The researchers initially informally contacted five participants in the study region via accidental sampling. This was conducted in March 2023. The trained research assistants conducted interviews to determine the participants’ relevance to the study. They asked questions about the participants’ ethnicity, residence location, and duration of residency in the study region. This further contributed to the establishment of trust and rapport with the initial five participants. The initial participants subsequently recommended the interviewees to other participants, who also provided significant insights relevant to the study. However, the study encouraged the initial participants to introduce the interviewee to individuals with diverse perspectives and experiences related to the study’s topic. The study also tracked the relationships and referral chains among participants to facilitate the final analysis of the sample composition. The initial participants (five) contacted for the study introduced the interviewees (trained research assistants) to an additional nineteen participants, and they willingly agreed to participate in the study. The interviews with these individuals took place between April 2023 and May 2023. The participants exhibited a diverse variety of opinions and experiences relevant to the study. The relationship between the initial participants and the individuals that they recommended for the study was mostly based on friendship and family ties.

#### 3.3.1. Inclusion and exclusion criteria.

The study involved adult males living in Kokrobite, Ghana, who were 18 years old or older and were single, married, separated, or cohabiting. Additionally, all participants must identify as Ga and be native Ga speakers. They also have to be from Ghana.

### 3.4. Data collection

The study gathered data through key informant interviews, in-depth interviews, and focus group discussions. Given that the study participants were only male respondents, the researchers trained three male teaching assistants to assist with data collection. The teaching assistants were solely male because many female researchers find it repressive, embarrassing, and emotionally demanding to interview males [24]. Additionally, since factors such as gender, class, and race also influence the research process [25], ensuring that the trained teaching assistants were exclusively male was crucial to address and overcome these challenges. The researchers also anticipated that some of the participants may experience emotional distress from narrating unpleasant past experiences. Therefore, when such issues arose, the researchers terminated the interviews and provided counseling by one of the principal researchers (RH), who possesses a psychology background. This allowed for prompt attention to these participants, as qualified counselors were not readily available within the study area. During the study, three instances of such occurrences arose, and the participants were provided with counseling. The information they provided was later omitted from the analysis.

The interviews were conducted in the Ga language, as it was the first language of the participants and they were proficient in speaking with it. Verbal consent was obtained from the participants before the interview, and it was also tape-recorded. The institutional review board approved this consent and this was witnessed by the research assistants engaged in the study. The duration of each interview ranged from 45 minutes to 1 hour. A sample of the questions in the interview guide encompassed questions on the respondents’ age, profession, present marital status, and number of children. We interviewed the participants about the attitudes of their community members towards an infertile man, the reactions of their family members and/or siblings towards a sibling who is unable to have a child, the capacity of childless men to take on leadership roles in the family or community, and the implications for childless lineages or family heads in carrying out various rites of passage. Field notes were taken after each interview, in addition to the tape recording, to assist the researchers in capturing valuable observations that were not recorded during the interviews.

#### 3.4.1. Key informant interviews.

The “Mankralo” and two family heads who are also from the Kokrobite area were among the three key informants that were also contacted. The “Mankralo” is a person with significant power who is responsible for communicating information about community events to the people. The purpose of the interview with these informants was to provide the researchers with valuable insights into the dynamics of marital relationships in Kokrobite, specifically focusing on the impact of infertility on these relationships, the consequences of a woman having a child outside of marriage, and the ability of childless men to assume leadership roles within the family or community. Finally, it is important to understand the fate of childless lineage or family heads in terms of their participation in different rites of passage. The principal researchers conducted these interviews, not the trained research assistants. The researchers obtained verbal consent from the key informants before the interview. We also used an audio recorder to record the entire interview. The researchers stopped the tape recording interview to discuss whether childless males could assume leadership positions in the family or community, as the three key informants felt more comfortable discussing this topic off-record. The time of each interview varied between 45 minutes and 1 hour. The researchers wrote field notes after each interview and used tape recording to capture significant observations not captured during the interviews. The key informant interview was conducted in May 2023.

#### 3.4.2. Focus group discussions.

The principal researchers conducted a focus group discussion based on the interview with the key informants. The primary purpose of the focus group guide, which comprised three questions, was to delve deeper into the topic of what happens concerning the performance of various rites of passage by lineage or family heads who do not have children. The significance of this topic lies in the fact that three key informants, during off-recording discussions, presented significant thoughts that require further discussion and interpretation from the standpoint of the local residents. The focus group guide also highlighted the actions taken by married males when they are unable to have children in their marriage, as well as the potential remedies for couples facing infertility.

One focus group discussion (FGD) was arranged and conducted in Ga. The focus group discussion was conducted by purposefully selecting nine male participants from the pool of 24 male participants who underwent interviews in the study. The nine participants consisted of two men who were cohabiting, three who were separated, three who were married, and one who was single. This was done to assist the researchers in having an integrated group for the discussion. Verbal agreement from the participants was obtained before the focus group discussion, which took place in May of 2023. The interviewees provided the participants with a set of criteria that highlighted their autonomy to express their opinions, even if they differed from those of others. The interviewers explicitly conveyed to the participants that there were no objectively correct or incorrect answers. The study recorded the focus group discussion with the participants’ verbal consent. The focus group discussion lasted approximately 1 hour and 30 minutes in total.

### 3.5. Data analysis

The interviews and focus group discussions were transcribed verbatim following meticulous listening to the recordings. This task was accomplished by the two principal researchers (GBA and RAH) and the three research assistants (FAB, DA, and JA), all of whom were proficient in the Ga language. Following transcription and verification, the transcripts were uploaded into NVivo, computer-assisted software used for managing and analyzing qualitative data, to conduct thematic analysis. As a result, inductive coding was performed. The two primary researchers (GBA and RAH) conducted an independent analysis of the transcripts and gave codes, which are concise phrase designations, to select parts of data that indicated their importance. The approved codes were subsequently applied for coding the remaining transcripts, which were then compared to ensure uniformity among the researchers. Any discrepancies were handled through discussions between the primary researchers (GBA and RAH) and the three research assistants (FAB, DA, and JA) until a consensus was achieved about the list of codes. As a result, the list was reviewed and the codes were grouped to create categories. Subsequently, these categories underwent a thorough evaluation in terms of their interconnection, and as a result, overarching themes were formed to accurately represent the significance of the collected data. The researchers utilized this newly developed coding structure to analyze and categorize the data obtained from the transcripts. The field notes collected throughout the study were evaluated and included in the process of analysis to enhance understanding and clarification of the data. The reliability of the findings was verified by reaching out to 10 interview participants, one family head, and three participants from the focus group discussion. This was done to validate the originally reported results as part of the participant validation procedure. The names of the participants presented in the findings were anonymized and given pseudonyms.

### 3.6. Ethical approval

The University of Ghana’s Ethics Committee for the Humanities (ECH) granted ethical approval for this study (reference: ECH 050/ 22–23), and informed consent was obtained from all participants who took part in the study.

## 4. Results

The study sample consisted of 27 male participants, ranging in age from 21 to 44 years old. The majority of the participants consisted of eleven masons and nine fishermen. Additionally, five participants worked as welders, 2 participants who worked as carpenters, and one unemployed participant (see Table 1). The majority of these participants were in a cohabiting relationship (12), married (6), or separated (5), while only a few were single (4). The male participants also varied in the number of children they possessed, ranging from three to six, with only one male participant not having a child. The results of the study can be categorized into five primary themes concerning male infertility: masculinization of adolescent males, blame for infertility, assessment of fertility (virility), repercussions of male infertility, and remedies for male infertility.

**Table 1:** Population characteristics.

Name	Age	Occupation	Level of Education	Current Marital Status	Number of children
Nii Kwartei	26	Mason	JHS	Cohabiting	3
James	37	Mason	SHS	Separated	5
Odartey	33	Fisherman	JHS	Separated	5
Nii Adu	30	Mason	JHS	Married	3
Tettey	35	Carpenter	SHS	Married	4
Kpakpo	28	Mason	JHS	Cohabiting	3
Allotey	32	Fisherman	JHS	Cohabiting	4
Nettey	40	Fisherman	JHS	Separated	6
Theo	32	Welder	No Education	Cohabiting	5
Ayikwei	25	Carpenter	JHS	Married	4
Addo	29	Fisherman	JHS	Married	3
Nii Amoo	33	Mason	No Education	Cohabiting	5
Abbey	31	Mason	JHS	Cohabiting	4
Nii Laryea	36	Fisherman	JHS	Separated	6
Ayitey	42	Welder	No Education	Cohabiting	5
Kwei	44	Welder	JHS	Married	4
Mantey	39	Fisherman	JHS	Cohabiting	3
Adolf	30	Mason	JHS	Married	3
Nii Commey	24	Unemployed	JHS	Single	2
Nii Darko	21	Mason	SHS	Single	–
Ayi	32	Fisherman	JHS	cohabiting	4
Sowah	41	Mason	SHS	separated	2
Adjetey	40	Welder	SHS	cohabiting	3
Lamptey	43	Fisherman	JHS	single	4
Okine	38	Mason	No Education	single	–
Lartey	41	Mason	JHS	cohabiting	5
Clottey	37	Welder	JHS	cohabiting	4

**JHS – Junior High School (**low educational status for junior high school and below**); SHS – Senior High School (**middle educational status for high school graduates**); No Education–** participant has no level of education.

### 4.1. The making of men

This theme highlights the cultural norm among the Ga people that promotes early fatherhood, with males starting to have children during adolescence. At this stage of an adolescent’s life, the Ga culture believes that a male child has reached an age where he can start engaging in sexual activities. The commencement of sexual activity also signifies that the male child has reached an age at which he is capable of procreating. Consequently, the study observed that adolescent males, as young as 15 years old, begin having children without being married. Members of this society do not frown upon fathering a child before marriage. The only downside is that it has the potential to disrupt one’s academic pursuits. Thus, parents frequently express discontent with their adolescent children who impregnate a girl while still attending school. Nevertheless, the importance and value attributed to children render the practice socially acceptable. Participants explained that it was common in the locality to take on an apprenticeship after completing one’s basic education. During this period, the male child is capable of trying to support himself with the meager income he obtains from his apprenticeship. At this stage, it is believed that getting a woman pregnant will make a man more responsible and force him to continue working hard and staying focused. This is due to the presence of an additional dependent in the form of his unborn child, which will require extra funds for sustenance. Therefore, it is unsurprising to witness young guys becoming fathers. The phenomenon is so widespread that young men who postpone fatherhood receive advice and guidance encouraging them to start a family. In the words of one participant:

*“I remember about 5 years ago, while I was learning masonry from my master, he was continuously pushing me to have kids. He said that I needed to prove my manhood in order to be considered a man. He even once placed a bet with me that he would cover the medical expenses of any woman I was able to get pregnant.” (Nii Darko, 21 years old, Single, Mason, no children)*

*“We the men in our town will not allow you to live in peace if, by the age of 20, 22, you are still single. For what more could you possibly be waiting for if you wait until you are in your 30s or 40s and still don’t have a child? When are you going to have a child and be able to care for them through school and other things? You won’t have the strength to work and make money if you wait too long, which makes it impossible for you to care for a child. You will come to the realization that without anybody to care for and work for, you cannot possibly be serious in life.” (Nii Laryea, 36 years old, fisherman, separated, 6 children)*

One result of the expectation from society to show one’s masculinity through sexual activity and childbirth is that some young men engage in promiscuous behavior or have several sexual partners. Peers ridicule young men who fail to express their masculinity through affairs, attributing their limited sexual activity to a hernia.

As a result, the conflation between impotence and infertility is not surprising. A common response to the question of what the community believes happens to infertile men or men who are unable to conceive was that being infertile meant that a man could not satisfy a woman sexually.

*“When you push these men, they won’t go. He’s running away if a woman clutches him. He will be quite hesitant to go join his wife in the bedroom when it is night-time and he has to lie down on the bed next to her, which will keep him locked in the living room sofa long into the night. His attitude will eventually drive the woman/wife to end the relationship.” (Allotey, 32 years old, fisherman, cohabiting, 4 children)*

*“Yeah, this is not at all unusual. Some guys avoid women because they know they are not good in bed. Occasionally, a woman may exhibit signals of interest in having a sexual relationship with a certain man, but he may choose to ignore her overtures to avoid embarrassing himself by admitting he has a problem (referring to poor sexual performance or impotence).” (Mantey, 39 years old, fisherman, cohabiting, 3 children)*

### 4.2. Blame for infertility

This section presents an analysis of the key individuals within the infertile man’s circle who end up being accused of being the reason behind the childless union. Frequently, people hold the spouses and mothers of men responsible for their infertility. Interestingly, the blame also often came from the man’s mother and extended family members and not necessarily the man himself. The quotes that follow further explain this:

*“Typically, women are held responsible for infertility, despite the fact that this should not be the case. If a couple is unable to have children in a marriage, it is recommended that the males leave the woman and marry another woman.” (Nettey, 40 years old, fisherman, separated, 6 children)*

*“For example, my elder brother had a child ten years ago but has been unable to have one again. Our mother seems to be blaming his wife for not having more children, as she is constantly telling her that she ought to have more. She doesn’t bother my brother about it.” (James, 37 years old, mason, separated, 5 children)*

*“Assuming responsibilities in a childless marriage might be challenging for a man. The men remain quiet while the family members cast blame upon the woman. Just one out of the lot is able to defend their wives.” (Nii Adu, 30 years old, mason, married, 3 children)*

Some participants even brought up the possibility that the man’s mother could be to blame. As a matter of fact, there are some men who hold their mothers responsible for their failure to have children or for whatever difficulties they encounter in their quest to have children. A participant expressed this:

*“Occasionally, the responsibility can also lie with the man’s mother. It is possible that his mother has transformed him into a woman. That is what some mothers do.” (Kwei, 44 years old, welder, married, 4 children)*

This assertion carries spiritual implications. It refers to a scenario in which the accused has engaged in rituals of faith that render their male child unable to fulfill his obligations and responsibilities as a male, including his reproductive function of producing sperm to fertilize his partner’s eggs.

Moreover, the men stated that, in certain cases, they must inquire with their parents to ascertain the reason behind their infertility and the inability to have children. Theo expressed this sentiment with the following words:

*“It is necessary for you to approach your mother or father and inquire about the reasons behind your existence, as well as the disparity between you and your peers in terms of having children. Despite engaging in sexual intercourse with a woman, you have observed that she does not conceive. Is the blame attributed to you or to the woman? Your father can inform you that your wife’s blood is incompatible with yours, which is the reason for your infertility. Therefore, unless you terminate your relationship with her, you will not be able to conceive a child.” (Theo, 32 years old, welder, cohabiting, 5 children)*

This demonstrates the important role that the community’s elders—in this case, the parents—play as the sources of wisdom that one should consult when seeking advice on how to resolve a problem.

### 4.3. Assessment of fertility

The results show that men in this society use traditional approaches to determine whether or not they are fertile. The first option that participants gave was a common method of assessing their reproductive potential. It’s not uncommon to find men having extramarital affairs as a way to prove their virility when they are unable to have children in their marriage. This is a result of the men’s reluctance to accept that they might be the reason for the infertility in their marriage. Hence, in an attempt to disprove this, he has extramarital affairs with at least two, three, or even four different women.

As per Odartey and Nii Laryea’s affirmation,

*“In order to ascertain whether the problem is with me, I will engage in sexual intercourse with two or three distinct ladies for a period of time. Typically, after a certain period, you will observe that one of them will approach you to convey the news of her pregnancy. It implies that you were not the primary cause of the problem at hand.” (Odartey, 33 years old, fisherman, separated, 5 children)*

*“I will engage in intimate relationships with other women due to the possibility of a mismatch between my wife’s blood and mine, which may prevent us from conceiving a child together. Should you choose not to go test on other ladies, how will you know? Therefore, it’s crucial that you double-check and be sure.” (Nii Laryea, 36 years old, fisherman, separated, 6 children)*

Furthermore, the attitudes conveyed by the participants depict the medical field as a final resort or, in certain instances, not a viable choice for males facing such circumstances.

*I will not visit the hospital. I want to ascertain on my own whether the problem truly comes from me. I will engage in sexual intercourse with my ‘jolly’ (girlfriend) or any woman of my choice, as I feel the need to assess and determine what options are available. There is no necessity to go to the hospital for any reason. (Kpakpo, 28 years old, mason, cohabiting, 3 children)*

This is due to the fact that men often do not believe that they are the reason for infertility in a relationship. For some, this view stems from their partners using contraceptives without their knowledge, and the misconception that extended use of these contraceptives can lead to infertility. According to a respondent,

*Some women are bad! They have taken an injection to prevent conception since they don’t want a child, even though you will be trying to get them pregnant in the interim. When you question them, they won’t even tell you. They will not confess to you until you have instilled fear in them. It is crucial that you question her since it’s possible that your wife is using a contraceptive, in which case you will be worried needlessly. (Nii Amoo, 33 years old, mason, cohabiting, 5 children)*

*Some injections administered to women may have adverse effects on their health. They will be receiving long-term injections as a contraceptive measure. Subsequently, once they cease taking any of the medication due to their desire to conceive, you will observe that they will encounter difficulties in getting pregnant. If she has been unable to get pregnant for a while, it is possible that the injections have produced problems in her body. I must talk to her about this problem. While some women will openly share their thoughts, others may feel embarrassed and hence choose to remain silent. However, I will ask her since I desire to make sure that the problem does not come from me. (Allotey, 32 years old, fisherman, cohabiting, 4 children)*

Men seeking to deny any apparent anomaly in their reproductive system that could be causing infertility in the partnership are viewed as attempting to prove their fertility to society.

### 4.4. Consequences of male infertility

In a patrilineal society, men hold significant leadership positions during crucial rites of passage, such as naming ceremonies, puberty rites, marriage rites, and funeral rites. It may be possible for a man who is the head of the lineage but is childless to assume the major duties during the rites of passage. This is often the case when the man shows his ability to be responsible by attending to the needs of other family members, such as by helping a niece or nephew obtain employment or by covering their school expenses. However, if he fails to demonstrate responsible behavior, he might lose the respect of the entire extended family and miss out on important traditional ceremonies. Society gossips about these men and criticizes them frequently. According to a participant,

*How do you spend all of your money? You are wasting yours on pointless things like taking care of dogs when humans cannot acquire food to eat and money to pay for their medical costs. (Nettey, 40 years old, fisherman, separated, 6 children)*

Again, the siblings of childless men often sympathize with their brother, who has not been able to have a child yet. These men receive psychological support and encouragement from their siblings. The men in the community also reported that, owing to the fact that the other siblings of the childless man have been able to bear children, the parents cannot push these men too hard, especially if their excuse is that they need grandchildren since their other children have been able to provide them with some. Rather, they simply advise and encourage their son, nephew, etc. about the importance of having their own biological child. In addition, the family does not become the subject of gossip from community members, as some of them have been able to bear children.

According to a respondent,

*My elder brother has been married for more than five years now without a child but I have not worried him about it. I don’t think it is necessary because my younger brother and I have about 6 children amongst us. But sometimes when our mother is worrying his wife about it, I pull him to the side that he should just try and find a solution to it to cut all this talking from our mother short…nobody worries me about my brother’s childlessness because they know I have children and my children are my brother’s children. (Adolf, 30 years old, mason, married, 3 children)*

Another consequence of being childless that was reported by participants is that, it does not establish your creditworthiness. This is due to the increased sense of responsibility that comes with having a child; it is unlikely for a man without children to receive a soft loan from a friend or family member. His perceived lack of financial responsibility stems from the fact that he has no dependents to provide for. The community in which he finds himself perceives him as lacking responsibility because he hasn’t developed financial discipline, which they attribute to not having children.

*On one occasion, I approached an elderly man asking for financial assistance. He responded directly and unequivocally, stating that he would not provide me with any money due to my lack of children and the perceived likelihood that I would squander it. He said that since my friend needs it to care for his child, he would prefer to give it to him. (Nii Darko, 21 years old, Mason, Single, No children)*

#### 4.4.1. Teasing/disgrace as a tool for encouraging fertility.

Additionally, the results of the study show that the adverse social responses experienced by an infertile male in his community serve as a mechanism to promote fertility. Therefore, it is essential to gain insight into responses that originate from the spouse, family members, friends, peers, and the general public concerning this issue. The results of the study show that its primary focus pertains to the notion of disgrace. Tettey, a married 35-year-old carpenter with four children, described the following:

*It is disgraceful to be a young male in the company of a group of friends who have all begun having children while you remain childless. You will develop a sense of isolation from your peer group.*

*Friends will make fun of you if you have no children whatsoever. They frequently tease you, and childless males are referred to as “kene” or “sekpe.” They will tease you whenever you are in their midst; you need to have a child; you need to have a child. It is truly abhorrent. (Okine, 38 years old, mason, single, no children)*

Labeling can therefore lead to shame. However, stigmatization does not accompany this experience. Thus, although men avoid becoming the target of such disgrace, they admit that society’s intentions are not to denigrate the individual, but rather to encourage fertility. As one respondent put it:

*For the youth in this community, we have two football teams, one for those who have children and the other for those who do not have any children. So, every now and then, these two teams play against each other. We do that to encourage those who don’t have children to learn from those who do and find a solution to their infertility. (Kpakpo, 28 years old, mason, cohabiting, 3 children)*

Friends of a man without children consistently express the need to have a child in their everyday interactions, often through playful teasing. The purpose of teasing is to serve as a reminder to these men that not having a child results in a lack of financial obligations and, thus, the forfeiture of some privileges within their social circle. For example, Kwei highlighted that:

*Even if we play a game and win and have to share the money, we don’t give those who don’t have children some of the money. We often tease them that ‘tsatsu tsɛ̃ɛ~ bo’ (they have no ants biting them) or ask them that, bo diɛŋtsɛ* *olɛeo Aduŋ? (Do you have a monkey you take care of?) and so they don’t need the money. (Kwei, 44 years old, welder, married, 4 children)*

*Sometimes, when we, the boys boys are sitting and having a chat, you will realize that someone will come and tell one person that his child is sick. This means he has to leave the group to attend to him. At that point, the attention of the group is drawn to the one who doesn’t have a child and then the subsequent conversation is directed at him: You too go and have a child; you too hurry up and have a child so that you will be more responsible. They make you feel very bad. (Nii Kwartei, 26 years old, mason, cohabiting, 3 children)*

### 4.5. Remedies for male infertility

The study also offered solutions for male infertility, and the respondents’ responses provided a range of strategies that infertile couples can employ to address the issue of infertility in their relationship. The participants in this section provide insights on the accepted cultural norms that guide a man in fulfilling the societal expectations of procreation, thereby ensuring his ability to meet this societal need.

First of all, according to Lartey, a 41-year-old mason with five children who lives with his partner:

*A woman can be allowed to leave the marriage if children are not forthcoming. It is a mutual understanding between both parties and she will not be reprimanded by family members as the need to perpetuate the lineage takes precedence. This is because it could be that her husband’s blood is not compatible with hers so they need to try with other partners.*

*Both the man and the woman can go out of the marriage in search of a child. However, this also marks the end of the relationship because a woman cannot bring a pregnancy from an extramarital affair into the marriage. (Ayitey, 42 years old, welder, cohabiting, 5 children)*

*In another instance,*

*A woman can have an extramarital affair on the blind side of her husband in order to get pregnant. She brings the pregnancy into the marriage and it solves the problem. However, if her husband later finds out about it, few of them accept it because he may have discovered along the line (through his own extramarital affairs, which did not yield any pregnancy) that he is the one with the problem. As such, he accepts the child as a means to hide his infertility from the public and to avoid being referred to as not man enough. (Nettey, 40 years old, fisherman, separated, 6 children)*

However, most men do not accept a pregnancy from an extramarital affair due to the dire consequences of disclosing their infertility, as well as their belief that they are not the cause of the infertility in the union. The consequences were expressed as:

*No, no, no, a man cannot accept a pregnancy from an extramarital affair. Everyone will get to know because, as the child grows, he will not resemble you… like the shape of his head or the way he walks or talks. In this town, everybody knows everyone, so it’s just a matter of time and we will all get to know who the true father of the child is through the child’s mannerisms and that is not good. It is an insult to the man and the family. Unless you go outside the community to have an extramarital affair, you cannot get away with it. You will, by all means, be caught. (Mantey, 39 years old, fisherman, cohabiting, 3 children)*

*It is risky to accept a pregnancy from an extra marital affair. You cannot trust a woman. One day in the heat of an argument, she will use it to insult you and say, ‘Bo diɛŋtsɛ* *nuu ji bo?’ (Are you a man?) And people will hear it and begin to ask questions about who the real father of the child is. (Nii laryea, 36 years old, fisherman, separated, 6 children)*

## 5. Discussion

This qualitative study explored community reactions to male infertility as experienced and reported by men within a patrilineal society in Ghana. According to the results of the current study, the early onset of childbearing, i.e., by adolescence, was a common practice, with older men in society encouraging young men to follow suit. People in the patrilineal community view this practice as a way to validate their manliness. This aligns with the findings from Worthman and Trang [26] and Mehra et al. [27], where young men perceive having a child early in life as a safeguard against irresponsibility.

The study also revealed that men’s mothers can bear the blame for infertility when their sons fail to conceive. This draws attention to the fact that the men in patrilineal society seem to continually exert their authority within the society, not only over their wives but also over their mothers, by seeing women as responsible for undesirable circumstances in their lives, such as infertility. The literature provides examples of how various parts of Sub-Saharan Africa have blamed wives for infertility [28–30]. However, the current study broadens the blame to encompass women related to the men in question, whether they are wives or mothers.

Additionally, it is believed that mothers use spiritual means to accomplish this feat. In traditional Ghanaian society, fertility gods have feminine identities in the form of river goddesses, tree goddesses, etc. [31]. It is believed that women are the custodians of fertility. It therefore makes sense that these same women have the power to withhold fertility if they so desire. This explains the belief by the men that their mothers have the potential to cause them to be infertile.

Studies in Africa have shown that one of the ways in which men resolve infertility in their marriages is to have an extramarital affair or to go in for another spouse [13,14]. The respondents in this study also reported similar behaviors. In addition, the study shows the ways in which men believe their spouses exhibit reproductive autonomy in their decision to use contraceptives, much to their chagrin. The findings of Mboane and Bhatta [32], Shakya et al. [33] and Adanikin et al. [34], which reported that women could not use contraceptives without their spouses’ consent, contradict this.

The findings of the study also revealed that the important role that men played in society as performers of rites of passage was not threatened if they did not have any biological children but, more importantly, if they played social fatherly roles, as the ability to fulfill the latter made up for their inability to fulfill the former. Bornstein et al. [35] report that infertile women either lose their rights to hold important positions reserved for them in society or cannot perform specific functions reserved for women with their own biological children. This underscores the advantages men possess over women in patriarchal societies. The findings also suggest that in some peri-urban Ghanaian communities, traditional ways of solving infertility through fostering are still socially acceptable.

The current study’s findings, which showed that peers in society teased and ridiculed infertile men (in the form of name calling otherwise referred to as labelling), are unique to this study. Furthermore, the unique aspect of this finding is the fact that the intention is to encourage such men to find a remedy to their situation rather than to victimize or make them feel isolated. The literature acknowledges that labelling is often accompanied by stigmatisation [36]. However, the findings in this study do not support that notion. With respect to the siblings of childless men, they played a laid-back role when it came to their reactions to infertility. They did not label their brother, and in a similar vein, society did not label them based on their family tie to the childless man. This is contrary to the discourses on labeling and stigmatization, where courtesy stigma, that is, stigma experienced by close ties of the target of stigmatization, was often reported by family members and close ties [37]. This is, however, surprising, as the current study argues otherwise.

The study highlights the significant role men continue to play in society as spiritual leaders, sought after by family members for spiritual guidance and advice. Traditionally speaking, a man’s role as head of the household, family, or lineage also came along with spiritual responsibilities. It was believed that because he was the eldest male member of the household, he was closest to the ancestors and could thus receive advice and direction from them when necessary. Therefore, when family members under his care faced any health challenge, he was the first to respond. It was his duty to seek answers from the ancestors and offer solutions to family members. The study population continues to recognize this crucial role in their quest for solutions to male infertility. Some respondents believed their parents, specifically their father, played an important role in solving their infertility by seeking a spiritual diagnosis for it, which inadvertently also provided a solution. Spiritual, not medical, consultations led to the discovery of blood incompatibility between spouses.

With regard to the concept of reproductive masculinity employed in this study, the findings point out that participants in this patrilineal society indeed saw themselves as virile, fertile, and capable of fathering their own children. In addition, they saw themselves as less susceptible to reproductive harm than women. According to these two beliefs, they would repeatedly switch partners to achieve the desired conception, blaming their wives or mothers for their marriage’s infertility and questioning their partners’ use of contraceptives to prevent pregnancy. Once again, the study’s findings broaden the discussion on reproductive masculinity by highlighting the potential loss of prominent roles for men without biological children in their communities. These men, who have proven themselves as responsible fathers by providing for the financial needs of their nephews, nieces, and other offspring within their extended family, do not face threats to their positions as heads of family, household, or lineage. They do indeed continue to hold prominent positions in society. However, the study participants did not exhibit the reproductive masculinity argument, which suggests that men are relatively distant from the health problems of the children they father. Rather, they highlighted the important role that fathers played when biological conception was not forthcoming. In cases of male infertility, they consulted these fathers, who provided spiritual diagnoses and explanations that addressed the issue of blood incompatibility between spouses and, consequently, the inability to conceive.

### 5.1. Limitations, future directions and implications

This qualitative study sought to shed light on the societal reactions to male infertility from the perspective of men in a patriarchal society. The men in this study, beneficiaries of power and authority in their social spaces, may express views influenced by their insider status (emic) and fail to consider the unequal position and experiences of women in their community. Furthermore, very few of the men in this study did not have children or were not experiencing male infertility. This means their narratives about societal reactions to male infertility stemmed from their position as outsiders, i.e., men who were not experiencing infertility. The fact that they are not the direct recipients of these reactions may dilute their accounts. However, this study’s focus group discussion provided a platform to hear the voices of the childless men and include them in the discussion. Future studies should consider the perspectives of women who may not be experiencing infertility in their relationships when examining societal reactions to infertility as well as men from higher socio-economic status who probably have different views on male infertility. Again, despite the study including one unemployed participant, future research should incorporate additional participants in similar circumstances to enhance discussions regarding social reactions to male infertility.

The findings of this study hold significant implications for policy and health care practice. It is critical that educational campaigns through the media and the Ghana Health Service focus on educating men, in particular, about the importance of seeking health care one year after trying to have a child and not being successful. Educating these men about their sexual and reproductive health is crucial for them to lead healthier and safer sexual lives.

## 6. Conclusion

This paper sought to discuss the societal reactions to male infertility in a patrilineal peri-urban community in Accra, Ghana. We discuss the discourses that go on amongst men as they make sense of their duties and responsibilities as men and fathers within the community. Participants espoused the importance of childbearing, viewing its early onset as a sign of men’s maturity. It was also important, as it helped a man to be more responsible and focused in life. Men with children, therefore, enjoyed subtle privileges within society that their counterparts without children did not. To encourage the men without children to begin childbearing, the men with children in this community teased such men as they went about their duties or during recreational activities such as football galas. Although childbearing was important to the men, it was also important that their partners did not bear children through extramarital affairs, as this would be a disgrace to them. When a marriage failed to produce children, it was acceptable for the man and woman to separate, as the cause of their infertility could be supernatural rather than biological. Again, this study found that men’s inability to have children did not threaten their leadership role during rites of passage, especially when they demonstrated or enacted social fatherly roles. We conclude that, to improve upon the fertility potential of men and to reduce the blame of infertility on women in patriarchal societies, there is a need to continuously sensitize men on infertility and the existence of male factor infertility such that they will be more open to seeking biomedical care.

## Supporting information

S1 File Interview guide. (DOCX)
