# Supplementary material for: ‘Tsatsu tsɛ̃ɛ̃ bo…’: Societal reactions to male infertility among the Ga’s in Ghana
Source: PLoS One. 2025 Aug 29;20(8):e0330529. doi: 10.1371/journal.pone.0330529 (PMC12396727; doi:10.1371/journal.pone.0330529)
Supplement: S1 File — (DOCX) [file pone.0330529.s001.docx]

**Appendix**

**Interview Guide**

**Section A**

Socio-demographic background of respondents

1. How old are you?
2. What is your marital status? Any children?
3. What is your religion?
4. What is your occupation?

**Section B**

Is there anything like infertility caused by a man? Explain your answer.

What happens to such men?

Describe the relationship:

1. In the house/home….
2. In the marital union….
3. Among his siblings
4. Among extended family members
5. Among his in-laws
6. In the community

**Section C**

What are the typical roles of the man (using the various rites of passage)

1. Outdooring
2. Puberty
3. Marriage
4. Funeral

How does male infertility interact/interfere with these roles?

What happens to the spouse/siblings/parents of such men in terms of their relationship with community members?

**Section D**

What solutions are available to such men in terms of:

1. Marriage
2. Childbearing
3. Status in society (family, community, church)

What are the consequences they face

1. In Marriage
2. In the extended family
3. Power/positions (status)
4. Amongst community members
